# Computerized Tomography Morphometric Assessment of the Internal Acoustic Meatus: Sex Differences, Orientation Angles, and Surgical Implications

**DOI:** 10.3390/jcm15031312

**Published:** 2026-02-06

**Authors:** Emine Deniz Gözen, Fırat Tevetoğlu, Ahmet Ertaş, Haydar Murat Yener, Osman Kızılkılıç, Ali İhsan Soyluoğlu

**Affiliations:** 1 Department of Otorhinolaryngology, Cerrahpaşa Faculty of Medicine, Istanbul University–Cerrahpaşa, Istanbul 34153, Turkey; emine.gozen@iuc.edu.tr (E.D.G.); hmuratyener@gmail.com (H.M.Y.); 2 Department of Anatomy, Cerrahpaşa Faculty of Medicine, Istanbul University–Cerrahpaşa, Istanbul 34153, Turkey; aertas@iuc.edu.tr (A.E.); ihsans@iuc.edu.tr (A.İ.S.); 3 Department of Radiology, Cerrahpaşa Faculty of Medicine, Istanbul University–Cerrahpaşa, Istanbul 34153, Turkey; osmank@iuc.edu.tr

**Keywords:** internal acoustic meatus, temporal bone, CT morphometry, lateral angle, skull base surgery

## Abstract

**Objective**: We aimed to evaluate the morphometric characteristics of the internal acoustic meatus (IAM) using high-resolution computed tomography (CT), with emphasis on sex- and age-related differences, with particular emphasis on the IAM orientation angle as a less-studied spatial parameter and its potential clinical and forensic relevance. **Methods**: Temporal bone CT scans of 162 patients (94 females, 68 males; age 1–77 years) were retrospectively analyzed. Measurements included the IAM inlet diameter, length, mid-diameter, lateral angle (LA), and orientation angle. Inter-observer agreement was assessed in 30 randomly selected cases. Morphometric parameters were compared by sex and age using *t*-tests and Mann–Whitney U tests. **Results**: Mean IAM lengths were 11.0 mm (right) and 11.1 mm (left), and the mean mid-diameter was 4.2 mm bilaterally. IAM lengths and diameters showed no significant sex- or age-related differences (*p* > 0.05). In contrast, LA and orientation angle differed significantly by sex (*p* < 0.05), with females showing higher LA values, which may influence posterior fossa surgical exposure. **Conclusions**: IAM size parameters are largely independent of sex and age, whereas lateral and orientation angles exhibit sex-related variation. Preoperative evaluation of IAM orientation on CT can support skull base surgical planning, and LA may provide supportive morphometric information in forensic contexts, although it should not be considered a standalone sex classification parameter.

## 1. Introduction

The internal acoustic meatus (IAM), located within the temporal bone, provides a connection between the inner ear and the posterior cranial fossa. It transmits essential neurovascular structures, including the vestibulocochlear nerve (cranial nerve VIII), the facial nerve (cranial nerve VII), the labyrinthine artery, and the vestibular ganglion. Understanding the anatomy of this region is not only crucial for anatomists, but also plays a significant role in the diagnosis and management of diseases involving the IAM.

Due to its complex morphology and the critical structures it contains, the IAM is of particular importance to both anatomists and surgeons. Comprehensive anatomical knowledge facilitates the interpretation of radiological imaging and helps prevent iatrogenic injuries during surgical procedures. The IAM can be involved in various pathological conditions such as tumors and vascular events, which may present with vestibular symptoms or hearing loss. Additionally, the compact and heat-resistant structure of the IAM allows it to remain preserved even in incinerated cadavers, highlighting its potential utility in forensic investigations, particularly for postmortem identification and sex estimation. With the increasing role of postmortem imaging and virtual anthropology, CT-based morphometric datasets are becoming an important complement to traditional osteological measurements.

Several studies have investigated the morphological characteristics of the IAM using radiological methods. Polat et al. assessed the length, width, and height of the IAM in CT images of healthy adults and reported significant differences in height between males and females, along with an age-related decrease in all dimensions after the age of 51 [1]. Markues et al. analyzed shape, area, aperture width, and longitudinal and vertical dimensions in individuals aged 1 to 92 years, and found statistically significant differences between pediatric and adult groups [2].

Among the morphometric features studied, the lateral angle of the IAM has attracted particular attention. Noren et al. found that the lateral angle could be useful in sex determination, even in incinerated cadavers, as its values correlated with the pelvic angle [3]. Gibelli et al. also observed a relationship between the lateral angle and IAM morphology, supporting its potential role in sex differentiation [4]. However, other studies have emphasized that this angle alone may not be sufficient for accurate sex estimation. Masotti et al. and Akansel et al. both concluded that while the lateral angle could serve as a supportive parameter, it should not be used in isolation [5,6].

Beyond anthropometric applications, the IAM has also been evaluated in the context of clinical outcomes. Giordano et al. examined the association between IAM geometry and hearing results in patients with vestibular schwannoma, reporting that larger IAM volume and diameter were linked to poorer postoperative hearing [7].

Although numerous CT-based studies have reported linear IAM measurements such as length and diameter, angular parameters describing its three-dimensional spatial orientation have been investigated far less frequently. In particular, the bilateral IAM orientation angle has not been systematically evaluated as a population-based morphometric variable. Therefore, the present study aims to assess both conventional IAM dimensions and orientation angles in a contemporary Turkish population, and to analyze potential differences according to sex and age, with consideration of their possible clinical and forensic relevance.

## 2. Materials and Methods

### 2.1. Study Design and Ethical Approval

This retrospective observational study was conducted in collaboration between the Departments of Anatomy and Radiology at Istanbul University-Cerrahpaşa, Cerrahpaşa Faculty of Medicine. Ethical approval was obtained from the Clinical Research Ethics Committee of the same institution (Approval No. 154388, dated 5 August 2021).

### 2.2. Study Population

Radiological images from 177 individuals aged between 1 and 77 years, acquired between August and November 2021, were retrospectively reviewed. Imaging had been performed for the evaluation of various otologic and cerebellopontine angle pathologies, including chronic otitis media, conductive or sensorineural hearing loss, facial nerve disorders, and vestibulocochlear nerve tumors.

Fifteen cases were excluded due to measurement inapplicability or lack of standardization, including unilateral IAM atresia (*n* = 1), petrous bone cholesteatoma (*n* = 1), intracanalicular vestibular schwannoma (*n* = 1), and inadequate reconstruction quality (*n* = 12). The final study group included 162 individuals.

### 2.3. Imaging Technique and Standardization

All high-resolution temporal bone CT scans were acquired using a 128-slice multidetector CT scanner (Siemens Somatom Definition AS+, Erlangen, Germany). Imaging parameters included 120 kVp, 200 mAs, 0.6 mm slice thickness, 0.3 mm reconstruction interval, pitch 0.8, and a 512 × 512 matrix. Axial images were reconstructed in the bone algorithm, and multiplanar reconstructions (MPR) were generated in axial, coronal, and oblique planes aligned to the lateral semicircular canal to optimize visualization of the internal acoustic meatus.

MRI was not routinely used for morphometric analysis. In selected cases with atypical IAM anatomy or suspected pathology on CT, MRI was performed using a 3.0 T scanner (Siemens Magnetom Skyra, Erlangen, Germany) with a dedicated head coil. The protocol included high-resolution axial and coronal T2-weighted sequences (slice thickness 0.6–0.8 mm) and contrast-enhanced T1-weighted sequences for intracanalicular assessment. MRI was used solely to confirm the integrity of the internal acoustic meatus and its contents and to exclude lesions that might affect CT measurements; no quantitative measurements were obtained from MRI.

To evaluate the reliability of the morphometric measurements, inter- and intra-observer agreement were assessed using the intraclass correlation coefficient (ICC). Two radiologists with 10 and 15 years of experience in head and neck imaging independently measured all IAM parameters in 30 randomly selected cases. One observer repeated the measurements after 2 weeks to assess intra-observer reproducibility. ICC values were calculated using a two-way random effects model with absolute agreement. ICC values were interpreted as follows: <0.50, poor; 0.50–0.75, moderate; 0.75–0.90, good; and >0.90, excellent reliability. All measurements demonstrated good to excellent agreement (ICC = 0.82–0.94 for inter-observer and ICC = 0.85–0.95 for intra-observer assessment).

### 2.4. Morphometric Parameters

**Figure 1:**
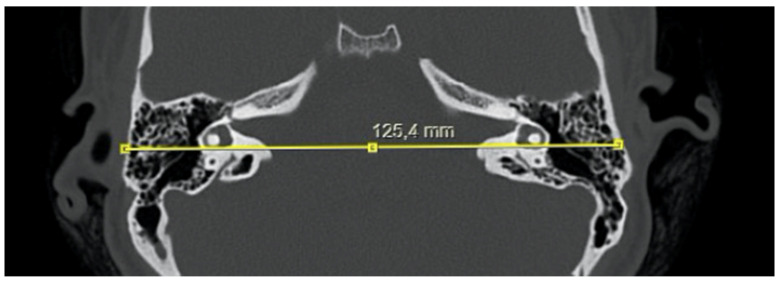
Axial high-resolution CT of the temporal bones showing measurement of the bitemporal distance (mm) using a line tangent to the lateral semicircular canals.

**Figure 2:**
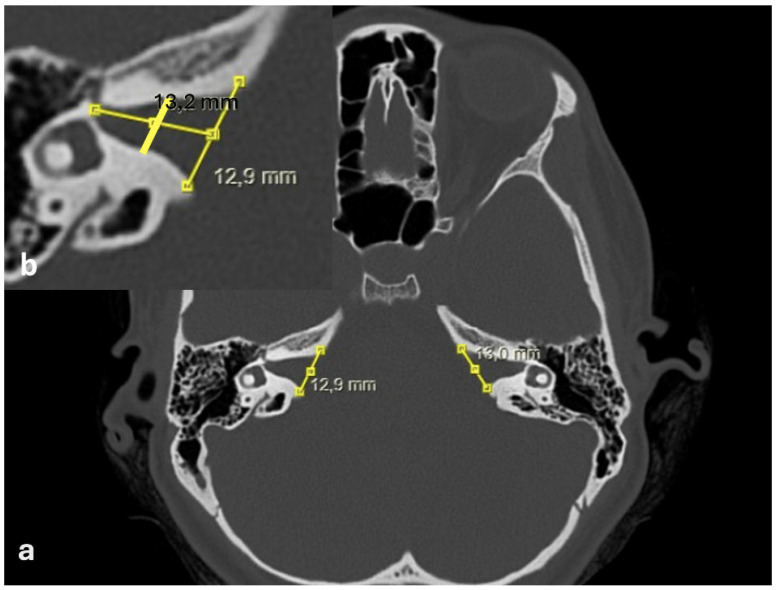
(**a**) Axial temporal bone CT image showing bilateral IAM inlet diameters (mm). (**b**) Measurement of right IAM length and mid-diameter (mm), with the line connecting the inlet midpoint to the most lateral point of the IAM.

**Figure 3:**
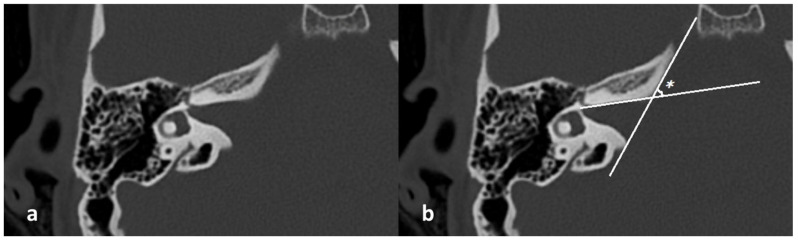
Measurement of the lateral angle (LA) on axial temporal bone CT. (**a**) Axial section at the level of the lateral semicircular canal and the geniculate ganglion. (**b**) Tangent lines (*) to the IAM inlet and anterior wall form the LA (°).

**Figure 4:**
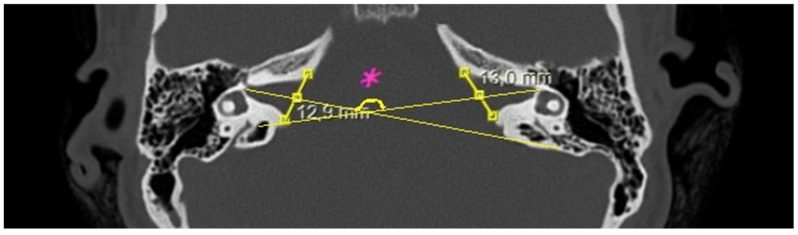
Axial temporal bone CT showing IAM orientation. The long axes of the IAMs (lines) form the orientation angle (°) (indicated with an asterisk), which may influence posterior fossa surgical exposure.

The following measurements were obtained from CT images:

- Bitemporal distance: the tangential line passing posterior to the lateral semicircular canals, measuring the distance between the cortices of both temporal bones (Figure 1);
- Bilateral IAM inlet diameter: the distance between the anterior and posterior walls at the most medial part of the IAM (Figure 2);
- Bilateral IAM length: the distance between the most lateral point of the IAM and the midpoint of the inlet diameter (Figure 2);
- Bilateral IAM mid-diameter: the distance measured at the midpoint of the IAM length, parallel to the inlet diameter (Figure 2);
- Right and left lateral angle: the angle between the line tangent to the IAM inlet and the line tangent to the anterior wall of the IAM (Figure 3);
- IAM orientation: the angle formed between the long axis lines passing through the midpoint of the inlet of each IAM (Figure 4).

All measurements were performed using the digital measurement tools of the CT workstation with images viewed in bone window settings. Care was taken to align the axial plane parallel to the lateral semicircular canal to ensure standardized orientation across subjects. Measurements were obtained at consistent anatomical landmarks to minimize observer-related variability. All measurements were statistically evaluated for variations according to age and sex.

### 2.5. Statistical Analysis

Statistical analyses were performed using the SPSS 20.0 software. Descriptive statistics were presented as mean, standard deviation, minimum, and maximum values for numerical variables, and as frequency and percentage for categorical variables. The distribution of the data was assessed using the Kolmogorov–Smirnov test. For comparisons, the independent samples *t*-test was used for normally distributed data, while the Mann–Whitney U test was applied for data that did not show normal distribution. A *p*-value of <0.05 was considered statistically significant. Effect size calculations and predictive modeling were not performed, as the aim of the study was descriptive morphometric assessment rather than diagnostic classification.

## 3. Results

Of the 162 cases included in the study, 94 (58.1%) were female and 68 (41.9%) were male. The mean age at the time of imaging was 38.9 years, ranging from 1 to 77 years.

In the overall group, the mean bitemporal distance was 126.3 mm (range: 89–147.4 mm). The mean right internal acoustic meatus (IAM) inlet diameter was 7.9 mm (range: 4.6–14 mm), the mean right IAM length was 11.0 mm (range: 7.1–16.6 mm), and the mean right IAM mid-diameter was 4.2 mm (range: 2.7–6.7 mm). The mean right lateral angle was 40.3° (range: 25.6–68.8°). For the left IAM, the mean inlet diameter was 8.1 mm (range: 4.2–12.2 mm), the mean length was 11.1 mm (range: 7.8–15.7 mm), and the mean mid-diameter was 4.2 mm (range: 2.7–6.1 mm). The mean left lateral angle was 40.6° (range: 23.5–71.6°). The mean IAM orientation angle was 155.6° (range: 121–182.3°) (Table 1).

In male cases, the mean age was 38.1 years (range: 1–77 years), and the mean bitemporal distance was 130.1 mm (range: 94.6–147.4 mm). The right IAM inlet diameter averaged 8.0 mm (range: 5–14 mm), the length was 11.6 mm (range: 7.9–16.1 mm), and the mid-diameter was 4.1 mm (range: 2.7–6.2 mm). The mean right lateral angle was 38.0° (range: 25.6–55.8°). On the left side, the mean inlet diameter was 8.0 mm (range: 5.9–11.3 mm), the length was 11.2 mm (range: 7.8–15.7 mm), and the mid-diameter was 4.3 mm (range: 3.0–6.1 mm). The mean left lateral angle was 38.9° (range: 25.6–57.4°), and the IAM orientation angle was 157.4° (range: 122.4–182.3°).

In female cases, the mean age was 38.7 years (range: 1–74 years), and the mean bitemporal distance was 123.6 mm (range: 89–136.6 mm). The right IAM inlet diameter was 7.8 mm (range: 4.6–12 mm), the length was 11.0 mm (range: 7.1–16.6 mm), and the mid-diameter was 4.2 mm (range: 2.7–6.7 mm). The mean right lateral angle was 41.9° (range: 26.7–68.8°). On the left side, the inlet diameter was 8.1 mm (range: 4.2–12.2 mm), the length was 11.0 mm (range: 8.2–15.2 mm), and the mid-diameter was 4.2 mm (range: 2.7–5.8 mm). The mean left lateral angle was 41.9° (range: 23.5–71.6°), and the IAM orientation angle was 154.3° (range: 121–181.2°).

Statistical analysis according to sex revealed significant differences in bitemporal distance, right lateral angle, left lateral angle, and IAM orientation (*p* < 0.05). However, no statistically significant differences were found in the bilateral IAM inlet diameters, lengths, or mid-diameters between male and female subjects (Table 2).

When evaluated according to age group (Group 1: ≤17 years; Group 2: >17 years), regardless of sex, a statistically significant difference was observed only in the bitemporal distance (*p* < 0.05). No significant differences were found in IAM inlet diameter, length, mid-diameter, lateral angles, or orientation between the age groups (Table 3)

## 4. Discussion

The internal acoustic meatus (IAM), located within the temporal bone, is an anatomical structure of considerable interest not only to anatomists but also to clinicians and forensic experts. In the present study, we assessed the orientation of the temporal bone, the anatomical position of the IAM, and potential morphological differences related to age and sex in a contemporary population living in Türkiye. Our findings are broadly consistent with the existing literature, which presents a range of IAM measurements obtained using different techniques and sample types.

Numerous studies have reported isolated morphometric measurements of the IAM. Farahani et al. measured the IAM in 14 temporal bones and found an average length of 8.4 mm, an anteroposterior (AP) diameter of 4.0 mm, and an opening width of 5.0 mm [8]. Mutlu et al., in a cadaveric study of 90 temporal bones, reported an average length of 10.98 mm [9], while Escajadillo, examining 50 adult specimens, found a shorter mean length of 6.9 mm [10]. A histological analysis of 435 temporal bones reported vertical and horizontal diameters of 3.68 mm and 3.72 mm, respectively [11]. Using silicone molds, Amjad et al. found a mean length of 9.9 mm and an AP diameter of 5.9 mm in 30 bones [12]. These diverse measurements align closely with the results obtained in our study.

CT-based investigations also yield similar findings. Valvassori et al. reported an IAM length of 8 mm and AP diameter of 4 mm in a cohort of 300 patients [13], while Day et al. found an average opening width of 5.9 mm using axial CT sections [14]. Three-dimensional CT reconstructions by Sakashita et al. revealed superior wall lengths of 10.6 mm and inferior wall lengths of 9.6 mm in individuals aged 1–72 years [15]. Another study using axial CT on 97 dissected temporal bones reported a mean length of 11.31 mm and AP diameter of 4.22 mm [16]. Fujita and Sando found a length of 11.5 mm and AP diameter of 3.6 mm in 108 bones [17], consistent with the 10.1 mm length reported by Silverstein et al. [18]. Krombach et al., using CT in 67 healthy subjects, found right and left IAM lengths of 11.34 mm and 11.33 mm, midpoint diameters of 4.43 mm and 4.55 mm, and opening widths of 7.39 mm and 7.49 mm, respectively [19]. Consistent with the demonstrated effectiveness and reliability in the literature, we also conducted our measurements on reconstructed images from axial CT sections. In our measurements of 162 cases, the mean right IAM opening width was 7.9 mm (4.6–14 mm), the mean right IAM length was 11 mm (7.1–16.6 mm), and the mean right midpoint diameter was 4.2 mm (2.7–6.7 mm). The mean left IAM opening width was 8.1 mm (4.2–12.2 mm), left IAM length was 11.1 mm (7.8–15.7 mm), and left midpoint diameter was 4.2 mm (2.7–6.1 mm).

Age-based comparisons in previous studies also support our findings. McClay et al. evaluated 309 pediatric temporal bones using CT and found a mean opening width of 5.02 mm [20]. Lang reported mean lengths of 11.15 mm and 7.23 mm, and opening widths of 6.46 mm and 4.3 mm in adults and children, respectively [21]. Barreto et al. found slight differences in opening width, length, and AP diameter between children and adults using microsurgical dissection (7.53 vs. 7.10 mm, 11.17 vs. 9.84 mm, and 4.82 vs. 4.47 mm, respectively) [22]. In our analysis, no statistically significant differences were found in bilateral IAM inlet diameter, length, or mid-diameter between individuals aged ≤17 and >17 years, although a significant difference in bitemporal distance was observed (*p* < 0.05). It should be noted that the limited number of pediatric cases in our sample may have influenced these results. A future study with a more balanced age distribution may provide more definitive conclusions.

The IAM also holds anthropological value due to its protected location within the dense pars petrosa, which resists trauma and high heat [3]. In this context, the lateral angle (LA)—the angle between the IAM and the petrous surface—has gained interest in forensic sex estimation. The concept, originally introduced by Wahl [23], was later developed by Noren et al. and adapted for CT-based measurements by Akansel et al. [3,6].

Embryologically, the angle may be shaped by the spatial growth dynamics of the brain and base of the skull, which influence the ossification of hyaline cartilage surrounding the developing IAM [3]. Thus, skull morphology, including skull diameters and the configuration of the pars petrosa, likely contributes to sex-related differences in LA, affecting IAM placement [24,25]. However, aside from one recent study, publications examining both LA and IAM morphology are limited [4]. Our study evaluated both IAM parameters (length and diameter) and LA, and the correlation between these variables was also examined. As previously reported, the measurements are not dependent on overall skull size [26].

In our study, right and left LA values were significantly higher in females than in males. The mean right LA was 41.930° in females and 38.090° in males; the mean left LA was 41.970° and 38.900°, respectively. While no significant sex-based differences were found in IAM inlet diameter, length, or mid-diameter, the sex-based difference in LA was statistically significant (*p* < 0.05). These findings are in agreement with Noren et al., who demonstrated LA to be compatible with the pelvic angle in cremated cadavers [3]. Masotti et al., in two separate studies, emphasized that LA alone was insufficient for sex estimation, although it may be useful in individuals under 70 years [5,27]. Akansel et al., the first to apply LA analysis to CT images, similarly concluded that LA should be used as a supportive—not definitive—parameter in sex determination [6]. Gibelli et al. measured IAM width, length, and angle in CT images of 100 individuals aged 20–70 years and found LA to be associated with IAM morphology and useful in sex differentiation [4]. Our results reinforce this position. However, despite statistical significance, the distributions of LA and orientation angle values showed partial overlap between males and females. Therefore, these parameters should be regarded as supportive morphometric indicators rather than definitive tools for sex classification, and their forensic utility is likely to be limited when used in isolation.

**Figure 5:**
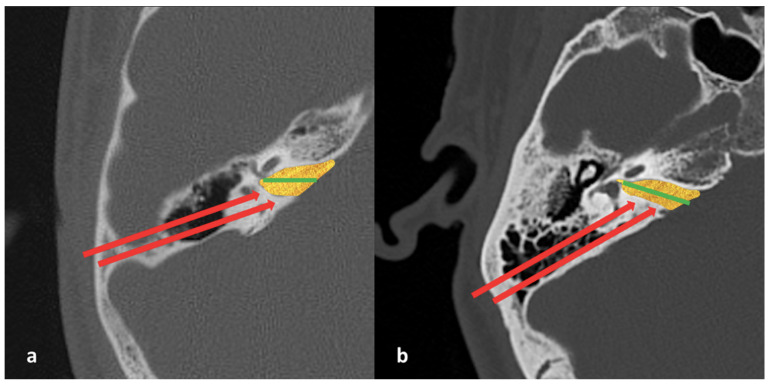
Impact of IAM orientation angle on surgical exposure during posterior fossa approaches. (**a**) Nearly 180°, providing a wider translabyrinthine view. (**b**) Less than 180°, resulting in relatively narrower exposure. (The yellow shaded area represents the internal acoustic canal (IAC). The green line indicates the orientation angle of the IAM. The red arrows illustrate the surgical access trajectory and exposure corridor toward the IAC.)

From a surgical perspective, the orientation and length of the IAM are also of importance. In translabyrinthine or minimally invasive transcanal transpromontorial approaches, IAM length and direction affect visualization and safe access to the facial nerve [28]. In the retrosigmoid approach, the horizontal orientation angle between the two IAMs determines surgical exposure: a narrower angle may facilitate access, while a wider angle could limit it (Figure 5). Although this aspect has not been previously addressed in the literature, our findings are informative. In our study, the mean IAM orientation angle was 155.6° (range: 121–182.3°); males had a mean angle of 157.4° (122.4–182.3°), and females had a mean angle of 154.3° (121–181.2°). The statistically significant sex difference suggests that posterior IAM exposure might be relatively more favorable in female patients.

Several limitations of this study should be acknowledged. The dichotomous age grouping and the relatively small pediatric subgroup may have reduced the sensitivity for detecting subtle age-related morphological differences in IAM morphology. In addition, although statistically significant sex-related differences were identified in angular parameters, no discriminant analyses, effect size calculations, or diagnostic accuracy assessments were performed; therefore, the forensic applicability of these measurements cannot be established from the present data. Furthermore, the proposed relationship between IAM orientation angle and posterior fossa surgical exposure is based on anatomical reasoning rather than direct surgical outcome data and should be considered hypothesis-generating. Finally, as the study reflects measurements obtained from a single-population cohort, population-specific craniofacial characteristics may influence IAM morphometry; therefore, extrapolation of these findings to other ethnic or demographic groups should be made cautiously. Future prospective studies integrating radiological morphometry with surgical correlation and forensic validation may help to better define the clinical and anthropological significance of IAM angular parameters.

## 5. Conclusions

The internal acoustic meatus (IAM) is a key anatomical structure that has attracted the attention of anatomists, clinicians, and forensic experts due to its complex morphology and clinical relevance. In this study, we investigated the morphological characteristics of the IAM within the temporal bone in a population residing in Türkiye, with a focus on age- and sex-related differences. Our findings demonstrated that, in agreement with the existing literature, there were no statistically significant differences in IAM inlet diameter, length, or mid-canal diameter between sexes or age groups. However, the lateral angle of the IAM was found to differ significantly between sexes and LA may provide supportive morphometric information in forensic contexts, but its discriminatory capacity is limited and should be used only in combination with other skeletal parameters. In addition, the orientation angle of the IAM, which also showed sex-based differences, may represent a hypothesis-generating anatomical observation of potential relevance for skull base surgeons, requiring further clinical validation. These results contribute to the morphometric understanding of the IAM in the Turkish population and may be useful in both clinical and forensic applications.

## Figures and Tables

**Table 1:** Demographic and morphometric characteristics of the study population.

	*n*	Minimum	Maximum	Mean	SD (±)
**Age**	162	1	77	38.94	19.69
**Bitemporal Distance (mm)**	162	89.00	147.40	126.38	9.41
**Right IAM Entry Diameter (mm)**	162	4.60	14.00	7.91	1.55
**Right IAM Length (mm)**	162	7.10	16.60	11.03	1.63
**Right IAM Mid-Diameter (mm)**	162	2.70	6.70	4.20	0.69
**Right Lateral Angle (°)**	162	25.60	68.80	40.31	8.01
**Left IAM Entry Diameter (mm)**	162	4.20	12.20	8.10	1.46
**Left IAM Length (mm)**	162	7.80	15.70	11.13	1.62
**Left IAM Mid-Diameter (mm)**	162	2.70	6.10	4.28	0.69
**Left Lateral Angle (°)**	162	23.50	71.60	40.68	8.92
**IAM Orientation (°)**	162	121.00	182.30	155.66	11.19

**Table 2:** Comparison of internal acoustic meatus morphometric measurements by sex (mean ± SD).

	Sex	N	Mean	Std. Dev.	*p*
**Age**	M	68	39.18	20.85	
	F	94	38.78	18.92	0.9 *
**Bitemporal Distance (mm)**	M	68	130.17	10.045	
	F	94	123.64	7.92	**0.001 ^§^**
**Right IAM Entry Diameter (mm)**	M	68	8.03	1.67	
	F	94	7.83	1.47	0.47 **^§^**
**Right IAM Length (mm)**	M	68	11.06	1.55	
	F	94	11.01	1.70	0.85 *
**Right IAM Mid-Diameter (mm)**	M	68	4.18	0.73	
	F	94	4.21	0.67	0.65 **^§^**
**Right Lateral Angle (°)**	M	68	38.09	6.80	
	F	94	41.93	8.46	0.002 *
**Left IAM Entry Diameter (mm)**	M	68	8.05	1.31	
	F	94	8.13	1.56	0.71 *
**Left IAM Length (mm)**	M	68	11.21	1.62	
	F	94	11.08	1.62	0.62 *
**Left IAM Mid-Diameter (mm)**	M	68	4.30	0.70	
	F	94	4.27	0.68	0.83 *
**Left Lateral Angle (°)**	M	68	38.90	7.60	
	F	94	41.97	9.59	**0.02 ^§^**
**IAM Orientation (°)**	M	68	157.46	10.98	
	F	94	154.36	11.22	**0.01 ^§^**

Bold values indicate statistically significant results. * Independent Samples *t*-Test. **^§^** Mann–Whitney U Test.

**Table 3:** Comparison of internal acoustic meatus morphometric measurements between pediatric (≤17 years) and adult (>17 years) groups (mean ± SD).

	Age	*n*	Mean	Std. Dev.	*p* Value
**Age**	≤17 years	27	8.33	5.47	
	>17 years	135	45.07	15.28	0.001 *
**Bitemporal Distance (mm)**	≤17 years	27	116.52	12.40	
	>17 years	135	128.35	7.29	**0.001 ^§^**
**Right IAM Entry Diameter (mm)**	≤17 years	27	7.64	1.97	
	>17 years	135	7.96	1.46	0.06 **^§^**
**Right IAM Length (mm)**	≤17 years	27	10.55	1.89	
	>17 years	135	11.13	1.57	0.145 *
**Right IAM Mid-Diameter (mm)**	≤17 years	27	4.34	0.63	
	>17 years	135	4.17	0.71	0.82 **^§^**
**Right Lateral Angle (°)**	≤17 years	27	42.52	6.91	
	>17 years	135	39.87	8.17	0.85 *
**Left IAM Entry Diameter (mm)**	≤17 years	27	8.20	1.38	
	>17 years	135	8.08	1.48	0.67 *
**Left IAM Length (mm)**	≤17 years	27	10.61	1.44	
	>17 years	135	11.24	1.63	0.06 *
**Left IAM Mid-Diameter (mm)**	≤17 years	27	4.44	0.65	
	>17 years	135	4.25	0.69	0.17 *
**Left Lateral Angle (°)**	≤17 years	27	40.30	9.30	
	>17 years	135	40.76	8.87	0.78 **^§^**
**IAM Orientation (°)**	≤17 years	27	153.05	9.50	
	>17 years	135	156.18	11.46	0.96 **^§^**

Bold values indicate statistically significant results. * Independent Samples *t*-Test. ^§^ Mann–Whitney U Test.

## Data Availability

The data presented in this study can be shared upon reasonable request from the corresponding author.
